# Poly(ionic liquid)s for Photo‐Driven CO_2_ Cycloaddition: Electron Donor–Acceptor Segments Matter

**DOI:** 10.1002/advs.202206687

**Published:** 2023-01-15

**Authors:** Xu Fang, Li Yang, Zhangben Dai, Die Cong, Daoyuan Zheng, Tie Yu, Rui Tu, Shengliang Zhai, Junxia Yang, Fengling Song, Hao Wu, Wei‐qiao Deng, Chengcheng Liu

**Affiliations:** ^1^ Institute of Molecule Sciences and Engineering Institute of Frontier and Interdisciplinary Science Shandong University Qingdao 266237 P. R. China; ^2^ State Key Laboratory of Molecular Reaction Dynamics Dalian Institute of Chemical Physics (DICP) Chinese Academy of Sciences Dalian Liaoning 116023 China

**Keywords:** CO_2_ utilization, donor–acceptor, metal‐free catalysts, photo‐driven CO_2_ cycloaddition, poly(ionic liquid)s, 148311, *N*,*N*‐dimethylformamide

## Abstract

CO_2_ cycloaddition with epoxides is a key catalytic procedure for CO_2_ utilization. Several metal‐based catalysts with cocatalysts are developed for photo‐driven CO_2_ cycloaddition, while facing difficulties in product purification and continuous reaction. Here, poly(ionic liquid)s are proposed as metal‐free catalysts for photo‐driven CO_2_ cycloaddition without cocatalysts. A series of poly(ionic liquid)s with donor–acceptor segments are fabricated and their photo‐driven catalytic performance (conversion rate of 83.5% for glycidyl phenyl ether) outstrips (≈4.9 times) their thermal‐driven catalytic performance (17.2%) at the same temperature. Mechanism studies confirm that photo‐induced charge separation is promoted by the donor–acceptor segments and can accelerate the CO_2_ cycloaddition reaction. This work paves the way for the further use of poly(ionic liquid)s as catalysts in photo‐driven CO_2_ cycloaddition.

## Graphical abstract

Electron donor–acceptor (D‐A) segments are introduced into poly(ionic liquid)s (PILs) for the first time to expand their applications in photo‐driven CO_2_ cycloaddition. The promoted separation of photo‐induced carriers by D‐A segments in PILs, together with the photo‐induced thermal effect, boosts the efficient photo‐driven CO_2_ cycloaddition without metals, cocatalysts, and solvents.

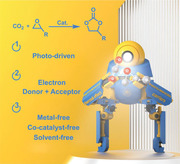

## 1 Introduction

The cycloaddition of CO_2_ with epoxides is an atom‐economical reaction without by‐products.^[^ ^1^, ^2^ ^]^ This reaction not only reduces the CO_2_ levels in the atmosphere but also enables CO_2_ conversion into high‐valued fine chemicals.^[^ ^3^ ^]^ The cyclic carbonates as products can be widely used as aprotic polar solvents, precursors of engineering plastics, and lithium‐ion battery electrolytes owing to their high boiling point, low toxicity, and high dissolution.^[^ ^4^, ^5^, ^6^ ^]^ However, almost all catalytic systems for CO_2_ cycloaddition require high temperature, high pressure, and long reaction time due to the high stability and low reactivity of CO_2_ molecules.^[^ ^7^, ^8^ ^]^ Such high energy input is always achieved through fossil fuels combustion, which accelerates the depletion of fossil fuels and increases greenhouse gas emissions. To this end, many research efforts have been devoted to developing low‐energy catalytic systems to make the CO_2_ conversion process more sustainable.^[^ ^9^ ^]^ It is particularly desirable to design catalytic systems that use renewable energy without consuming fossil fuels.^[^ ^10^ ^]^

Solar energy, as an inexhaustible renewable resource, can be used to overcome the activation barrier in CO_2_ cycloaddition with minimal environmental impact. So far, several catalysts (e.g., CoPc/TiO_2_, Bi‐Porous Coordination Network (PCN)‐224, Al—N—C, and HPC‐800) have been used for light‐induced CO_2_ cycloaddition with epoxides, in which metal species and nucleophilic cocatalysts are indispensable.^[^ ^11^, ^12^, ^13^, ^14^, ^15^ ^]^ Metal species and cocatalyst, e.g., tetrabutyl ammonium bromide (TBAB) can drive the epoxide ring‐opening, which is the rate‐determining step (RDS) for CO_2_ cycloaddition.^[^ ^12^, ^15^, ^16^, ^17^ ^]^ Yet, these metal‐based catalysts always require time‐consuming multistep preparation.^[^ ^16^ ^]^ Moreover, the separation of the metal species and homogeneous cocatalysts from products after reaction are time‐consuming and costly. In this regard, developing high‐performance catalytic system without metal species, cocatalysts, and solvents is highly demanded but challenging. Owing to their CO_2_ activation ability, ionic liquids and poly(ionic liquid)s (PILs) with ionic sites have been adopted as catalysts in thermal‐driven CO_2_ cycloaddition.^[^ ^16^, ^18^, ^19^, ^20^ ^]^ Nevertheless, the photoelectric properties of PILs are rarely studied and PILs for photo‐driven CO_2_ cycloaddition remain unexplored. It is reported that the ring‐opening of epoxides can be promoted by photogenerated carriers in catalysts.^[^ ^12^, ^13^ ^]^ Materials containing electron donor–acceptor (D‐A) segments, could promote the photoinduced carrier separation and transfer.^[^ ^21^, ^22^ ^]^ Accordingly, we hypothesize that the improved carrier separation efficiency in the PILs catalysts is vital to the high reaction activity, and thus the PILs structures with D‐A segments need to be elaborately designed. There has yet to be a report on the development of PILs catalysts with electron D‐A structures for CO_2_ cycloaddition.

Based on the preceding discussions, for the first time, we introduce electron D‐A segments into PILs as metal‐free catalysts for photo‐driven CO_2_ cycloaddition with epoxides. The efficiency of these PILs in photo‐driven CO_2_ cycloaddition outperforms the thermal‐driven efficiency at the same temperature. Specifically, the photo‐driven conversion of the glycidyl phenyl ether using T2‐PIL is 4.9 times of the thermal‐driven one. The synergistic effect of photo‐induced thermal effect and photo‐induced charge separation in PILs are responsible for the excellent performance. Density functional theory (DFT) calculations and electron paramagnetic resonance (EPR) spectra demonstrate that the photo‐induced charge separation promoted by the electron D‐A segments can significantly accelerate the CO_2_ cycloaddition reaction via electron transfer to the epoxide. Moreover, because triazine rings and imidazolium cations coexist as sufficient active sites in T2‐PIL, satisfactory conversion rates can be achieved without the use of cocatalysts or solvents, avoiding tedious postreaction treatment.

## 2 Results and Discussion

### 2.1 Structural Design of Poly(ionic liquid)s for Photo‐Driven CO_2_ Conversion

**Figure 1:**
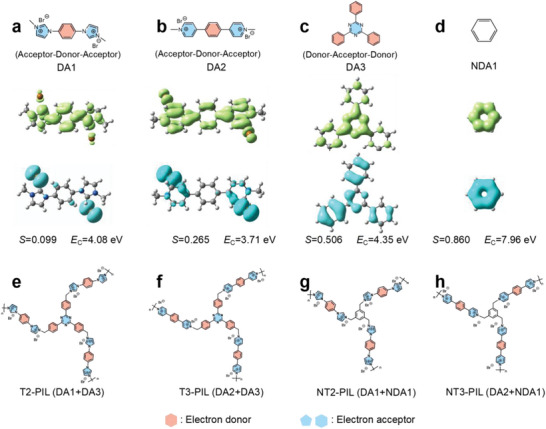
Theoretical design of PILs structures for photo‐driven CO_2_ cycloaddition. The electron (lime) and hole (cyan) distribution of S1 excited state on a) DA1, b) DA2, c) DA3, and d) NDA1 segments. *S* is the overlap integral of electron–hole distribution and *E*_C_ is the coulomb attraction energy between the hole and electron. e–h) Structures of PILs with different D‐A segments.

Herein we design two imidazolium‐based poly(ionic liquid)s (T2‐PIL and NT2‐PIL) and two pyridinium‐based poly(ionic liquid)s (T3‐PIL and NT3‐PIL) with D‐A segments for photo‐driven CO_2_ cycloaddition. First, time‐dependent (TD)‐DFT is used to explore the electron transfer in D‐A segments including DA1, DA2, and DA3 (**Figure** **1**a–c). The electron and hole distributions of the models are constructed by Multiwfn and visual molecular dynamics.^[^ ^23^, ^24^ ^]^ The electron–hole analysis module of Multiwfn has been widely used to do the electron excitation analysis.^[^ ^25^, ^26^, ^27^, ^28^ ^]^ In the electron–hole theory, the overlap integral (*S*) and the coulomb attraction energy between hole and electron (*E*_C_) values are usually applied to evaluate the electron–hole separation (the calculation details can be found in the Experimental Section). The smaller *S* and *E*_C_ indicate more evident electron–hole separation. As shown in Figure 1a,b, the ionic DA1 and DA2 all show small *S* and *E*_C_ (*S* for DA1 and DA2 are 0.099 and 0.265; *E*_C_ for DA1 and DA2 are 4.08 and 3.71 eV, respectively), which denotes good electron–hole separation. The triazine‐based DA3 shows moderate *S* (0.506) and *E*_C_ (4.35 eV) (Figure 1c). In contrast, the *S* (0.860) and *E*_C_ (7.96) of benzene ring are both large (Figure 1d), indicating that benzene ring is a non‐D‐A structure (NDA1). The above results indicate that DA1, DA2, and DA3 all can benefit the separation of hole and electron efficiently. Second, PILs with different D‐A segments are designed for photo‐driven CO_2_ cycloaddition. T2‐PIL and T3‐PIL both comprise two D‐A segments, i.e., DA1 and DA3 in T2‐PIL, DA2 and DA3 in T3‐PIL (Figure 1e,f). For NT2‐PIL and NT3‐PIL, DA3 is replaced by NDA1 (Figure 1g,h). Consequently, the electron–hole separation in T2‐PIL and T3‐PIL are expected to be more efficient than in NT2‐PIL and NT3‐PIL.

### 2.2 Synthesis and Structural Characterization of Poly(ionic liquid)s

The designed PILs are then synthesized by a one‐step Menshutkin reaction (Figure S1 and Table S1, Supporting Information). Take T2‐PIL and T3‐PIL as two examples, the successful preparations of PILs are confirmed by solid state ^13^C nuclear magnetic resonance (NMR) and Fourier transform infrared (FT‐IR) spectra. For the solid‐state ^13^C NMR spectrum of T2‐PIL (Figure S2a, Supporting Information), the distinct signals at 136 and 124 ppm can be assigned to the C1 and C2–C3 atoms in imidazolium cation rings^[^ ^29^ ^]^ and the peak at 170 ppm can be assigned to the carbon atoms in the triazine rings.^[^ ^30^ ^]^ For T3‐PIL (Figure S2b, Supporting Information), the distinct signals at 146, 130, and 155 ppm can be assigned to the C1, C2, and C3 atoms in pyridinium cation rings, respectively.^[^ ^31^ ^]^ The peak at 171 ppm can be assigned to the carbon atoms in the triazine rings. According to the FT‐IR spectrum of T2‐PIL (Figure S2c, Supporting Information), the absorption peaks located at 1659, 1564, 1108, and 1075 cm^−1^ are characteristic peaks of the stretching vibration of the imidazolium ring.^[^ ^32^ ^]^ The peaks at 1510 and 1361 cm^−1^ are characteristic peaks of the triazine ring, which also appear in the spectrum of T3‐PIL (Figure S2d, Supporting Information).^[^ ^33^ ^]^ In addition, an absorption peak at 1628 cm^−1^ can be observed for T3‐PIL (Figure S2d, Supporting Information), attributing to the stretching vibration of the C=N^+^ bond in the pyridinium cation ring.^[^ ^34^ ^]^ X‐ray photoelectron spectroscopy (XPS) measurements are performed to determine the chemical composition and electronic structure of T2‐PIL and T3‐PIL. In N 1s XPS spectra (Figure S2e,f, Supporting Information), the peak of N 1s is deconvolved into two obvious peaks. The peak at about 399 eV can be attributed to the N 1s in the triazine ring and the peak around 402 eV can be attributed to the N 1s on imidazolium‐based cations and pyridinium‐based cations.^[^ ^34^, ^35^ ^]^ Moreover, the element contents in the PILs determined by XPS and oxygen bomb‐ion chromatography are close to the theoretical values. Thermogravimetric analysis shows that the decomposition temperatures of T2‐PIL and T3‐PIL are about 333 and 332 °C, respectively (Figure S3, Supporting Information). Scanning electron microscopy and transmission electron microscopy images (Figure S4, Supporting Information) show that T2‐PIL and T3‐PIL have completely different morphological characteristics.

**Figure 2:**
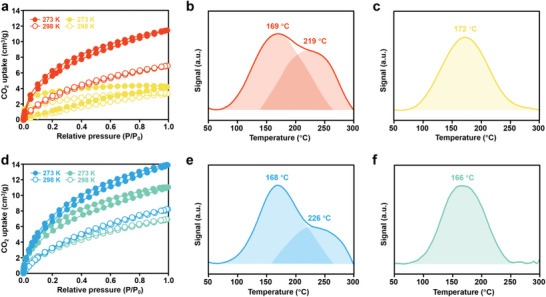
a) CO_2_ adsorption–desorption isotherms of T2‐PIL (red) and NT2‐PIL (yellow). Temperature‐programmed CO_2_ desorption measurement of b) T2‐PIL and c) NT2‐PIL. d) CO_2_ adsorption–desorption isotherms of T3‐PIL (blue) and NT3‐PIL (green). Temperature‐programmed CO_2_ desorption measurement of e) T3‐PIL and f) NT3‐PIL.

In the catalytic conversion of CO_2_, the density of binding sites and their binding strength with CO_2_ in catalysts are vital to their performance. Therefore, the CO_2_ adsorption capacity of the PILs is investigated by CO_2_ physical adsorption (**Figure** **2**a,d). T2‐PIL and T3‐PIL containing triazine groups exhibit much higher CO_2_ adsorption capacity than NT2‐PIL and NT3‐PIL without triazine groups. It suggests that the introduction of triazine rings in PILs can enhance their adsorption capacity for CO_2_.^[^ ^20^ ^]^ This is further confirmed by the CO_2_ adsorption test of CTF‐2‐400, which is rich in triazine groups and shows the highest CO_2_ uptake capacity (Figures S6 and S7a, Supporting Information). The binding strength of different binding sites in these PILs are further studied by temperature‐programmed CO_2_ desorption tests. T2‐PIL and T3‐PIL with triazine groups both exhibit two desorption peaks (Figure 2b,e), while NT2‐PIL and NT3‐PIL only exhibit one desorption peak (Figure 2c,f). The desorption peaks at lower temperatures (169, 172, 168, and 166 °C for T2‐PIL, NT2‐PIL, T3‐PIL, and NT3‐PIL, respectively) can be assigned to the imidazolium rings and pyridinium rings. The desorption peaks at higher temperatures (219 and 226 °C) can be attributed to the triazine rings in T2‐PIL and T3‐PIL, indicating their stronger basicity and interactions with CO_2_. These strong interactions are expected to bring about extraordinary CO_2_ activation ability.

To determine the intermediate species during CO_2_ chemisorption on PILs, we conduct in situ diffuse reflectance infrared Fourier transform spectroscopy (DRIFTS). First, we purge CO_2_ in T2‐PIL and NT2‐PIL for 5 min. Second, CO_2_ is cut off and the samples stay in static CO_2_ atmosphere. In the meantime, spectra are collected at different time intervals (Figure S8a,b, Supporting Information). For T2‐PIL, seven new peaks between 1700 and 1600 cm^−1^ emerge after introducing CO_2_ and get stronger with increasing time. While for NT2‐PIL, only one peak at 1660 cm^−1^ arises after 20 min, which can be attributed to carboxylate species forming on imidazolium rings.^[^ ^36^, ^37^, ^38^, ^39^ ^]^ Apart from the peak at 1660 cm^−1^, other peaks in the range of 1700–1600 cm^−1^ for T2‐PIL can be assigned to the carbonate species forming on the triazine rings.^[^ ^39^, ^40^ ^]^

### 2.3 Cycloaddition Reaction of CO_2_ with Epoxides

**Figure 3:**
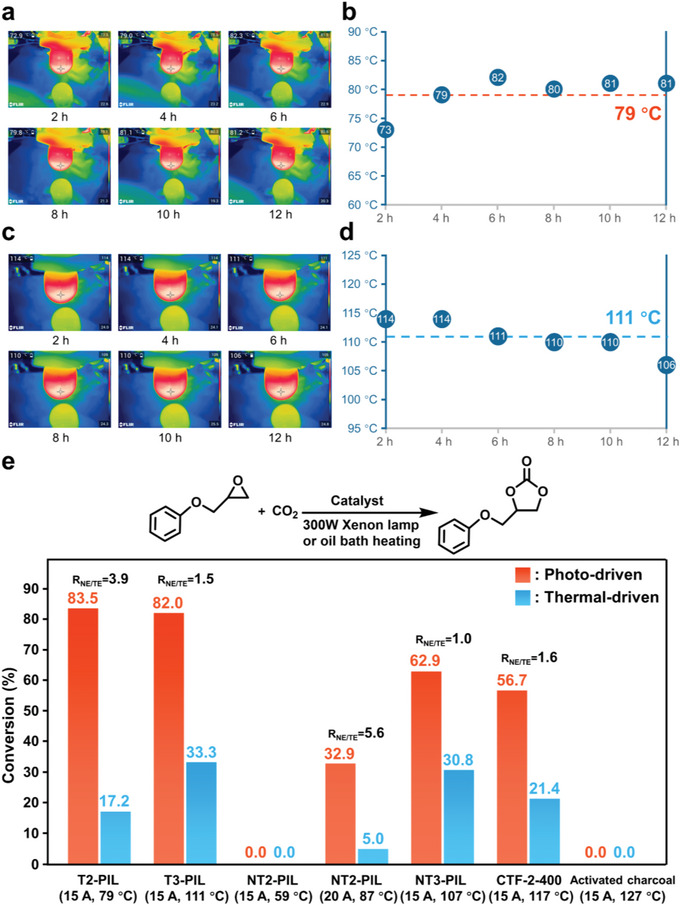
a) IR thermal images and b) temperature of the reaction system with T2‐PIL as catalyst under light irradiation. c) IR thermal images and d) temperature of reaction system with T3‐PIL catalyst under light irradiation. e) Conversion rate of glycidyl phenyl ether with different catalysts in photo‐driven and thermal‐driven CO_2_ cycloaddition (The values in parentheses represent the current of the Xenon lamp and corresponding temperature by light‐induced thermal effect).

The above results imply that both T2‐PIL and T3‐PIL are ideal candidates for CO_2_ capture and conversion. To make full use of solar energy, the light absorption properties of the PILs are investigated. It can be seen from UV–vis diffuse reflectance spectra that the light absorptions of the PILs mainly locate in 200–500 nm (Figure S9, Supporting Information). Hence full‐spectrum light is used in the photo‐driven reactions. Due to its large size, glycidyl phenyl ether is usually regarded as one of the most difficult epoxides to react in the reported studies, and higher temperature and longer reaction time are required.^[^ ^41^, ^42^ ^]^ Herein glycidyl phenyl ether is chosen as a model substrate for catalyst screening and reaction parameters optimization . In a typical catalytic experiment, only the catalysts and glycidyl phenyl ether are charged into the reaction vessel, and a CO_2_‐filled balloon is used as CO_2_ source. The system temperature is recorded by an infrared thermal camera during the photo‐driven reaction, and the average temperature is taken as the reaction temperature for thermal‐driven catalysis. Different catalysts exhibit different solution temperatures exposing to irradiation, e. g., 79 and 111 °C for T2‐PIL and T3‐PIL, respectively (**Figure** **3**a–d).

From the catalytic results (Figure 3e), both T2‐PIL and T3‐PIL exhibit high activities in photo‐driven CO_2_ cycloaddition (83.5% and 82.0% conversion rate with T2‐PIL and T3‐PIL, respectively). There is a massive leap over the thermal‐driven reactions in the dark (17.2% and 33.3% conversion rate with T2‐PIL and T3‐PIL, respectively) at the same temperature. For T2‐PIL, the conversion rate of glycidyl phenyl ether in photo‐driven reaction can reach 4.9 times of the thermal‐driven reaction. The ratio of nonthermal effect to thermal effect (*R*_NE/TE_) is used to evaluate the influence of nonthermal effect in photo‐driven processes.^[^ ^43^ ^]^ The *R*_NE/TE_ of T2‐PIL is 3.9, indicating that the nonthermal effect, i.e., light‐induced charges in T2‐PIL play an important role in this photo‐driven catalytic process. Even for thermal‐driven cycloaddition at 120 °C, the conversion rate (71.9%) is still lower than that of the photo‐driven catalytic reaction at 79 °C. To the best of our knowledge, the reaction rate (19.5 mmol g^−1^ h^−1^) and turnover frequency (TOF = 5.90 h^−1^) of glycidyl phenyl ether in the photo‐driven catalysis with T2‐PIL is one of the highest among both metal‐free or metal‐based heterogeneous catalysts without cocatalysts under atmospheric pressure (e.g., 8.03 mmol g^−1^ h^−1^ and 3.09 h^−1^ with PDMBr at 120 °C) (Tables S3 and S4, Supporting Information).^[^ ^44^ ^]^ Among the reported catalysts for photo‐driven CO_2_ cycloaddition, the *R*_NE/TE_ of T2‐PIL is also the highest (Table S5, Supporting Information). Other PILs with D‐A segments, i.e., T3‐PIL, NT2‐PIL, and NT3‐PIL, all show higher conversions in photo‐driven reactions than thermal‐driven reactions (Figure S3e, Supporting Information). These results indicate that the photo‐induced charges generated in these PILs significantly promote the CO_2_ cycloaddition.

CTF‐2‐400 also shows a moderate *R*_NE/TE_ of 1.6, indicating that nonthermal effect also exists in CTF‐2‐400 (Figure 3e).^[^ ^45^ ^]^ The lower conversion with CTF‐2‐400 at higher temperature (56.7% at 117 °C) than with T2‐PIL (83.5% at 79 °C) in photo‐driven reactions may result from the lack of ionic sites in CTF‐2‐400, which is important for promoting the ring‐opening of epoxides.^[^ ^46^ ^]^ We also use commercially available activated charcoal as a control catalyst, which can effectively convert solar energy into heat.^[^ ^13^ ^]^ The activated charcoal exhibits the highest solution temperature (127 °C) at the same light intensity, while without any activity (Figure 3e). This can be attributed to the absence of catalytic active sites in the activated charcoal. 1‐Octyl‐3‐methylimidazolium bromide (OmimBr‐IL) and TBAB with ionic sites are selected as two homogeneous catalysts (Figure S13a, Supporting Information).^[^ ^47^, ^48^ ^]^ Both of them exhibit the same conversions in photo‐driven and thermal‐driven catalytic reactions without nonthermal effect (Figure S13, Supporting Information), confirming that only the PILs with D‐A structure can promote electron–hole separation to accelerate this reaction. The TOF of glycidyl phenyl ether with T2‐PIL can reach 11 times that of OmimBr‐IL and 15 times that of TBAB in photo‐driven CO_2_ cycloaddition, validating the importance of D‐A segments. To verify the compatibility of the PILs, epibromohydrin, epichlorohydrin and allyl glycidyl ether are selected as three substrates and T2‐PIL all show high conversion and *R*_NE/TE_ in the photo‐driven reactions (Figure S14a, Supporting Information). T2‐PIL can maintain high activity after five cycles of experiments, indicating its good stability under light irradiation (Figure S14b, Supporting Information). The cycloaddition reactions under different light intensity are also conducted to investigate the effect of light intensity. It can be seen that stronger light intensities can bring about higher conversions (Figure S14c, Supporting Information).

### 2.4 Revalidation of Catalyst Design Strategy

**Figure 4:**
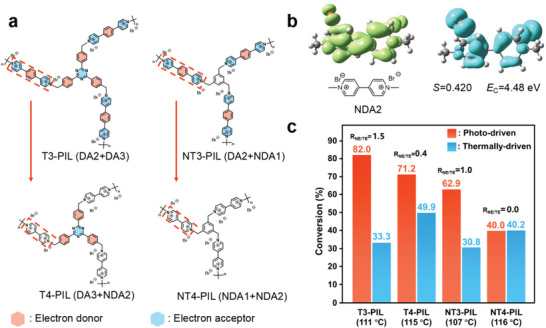
a) Structure design of PILs with non‐D‐A segments. b) The electron (lime) and hole (cyan) distribution of *S1* excited state on NDA2. *S* is the overlap integral of electron–hole distribution and *E*_C_ is the coulomb attraction energy between the hole and electron. c) Conversion rate of glycidyl phenyl ether with different catalysts in photo‐driven and thermal‐driven CO_2_ cycloaddition with the Xenon lamp current of 15 A (The values in parentheses represent the system temperature under irradiation).

To further validate the vital role of the D‐A segments in the photo‐driven CO_2_ cycloaddition, T4‐PIL with partial D‐A segments and NT4‐PIL without D‐A segments are designed and synthesized (**Figure** **4**). In T4‐PIL and NT4‐PIL, the D‐A segments DA2 in T3‐PIL and NT3‐PIL are substituted with non‐donor–acceptor fragment NDA2 (Figure 4a,b). TD‐DFT calculations are conducted to show the difference of electron–hole distributions between DA2 and NDA2 (Figures 1b and Figure 4b). As shown in the electron–hole calculation results, the overlap integral *S* of NDA2 (0.420) is much larger than that of DA2 (0.265), which denotes lower electron–hole separation degree in NDA2. The lager *E*_C_ in NDA2 (4.48 eV) than DA2 (3.71 eV) also supports this conclusion. Therefore, the photocatalytic performance of T4‐PIL (NT4‐PIL) is worse than that of T3‐PIL (NT3‐PIL) from the theoretic perspective. Then these two PILs are also used as catalysts in photo‐driven and thermal‐driven CO_2_ cycloaddition (Figure 4c). The experimental results show that the *R*_NE/TE_ is reduced from 1.5 to 0.4 when the catalyst changes from T3‐PIL to T4‐PIL. For NT4‐PIL without any D‐A segments, the conversion rate remains the same in photo‐driven and thermal‐driven reactions (40.0% and 40.2%, respectively), indicating the nonthermal effect even disappears completely.

### 2.5 Mechanistic Study

**Figure 5:**
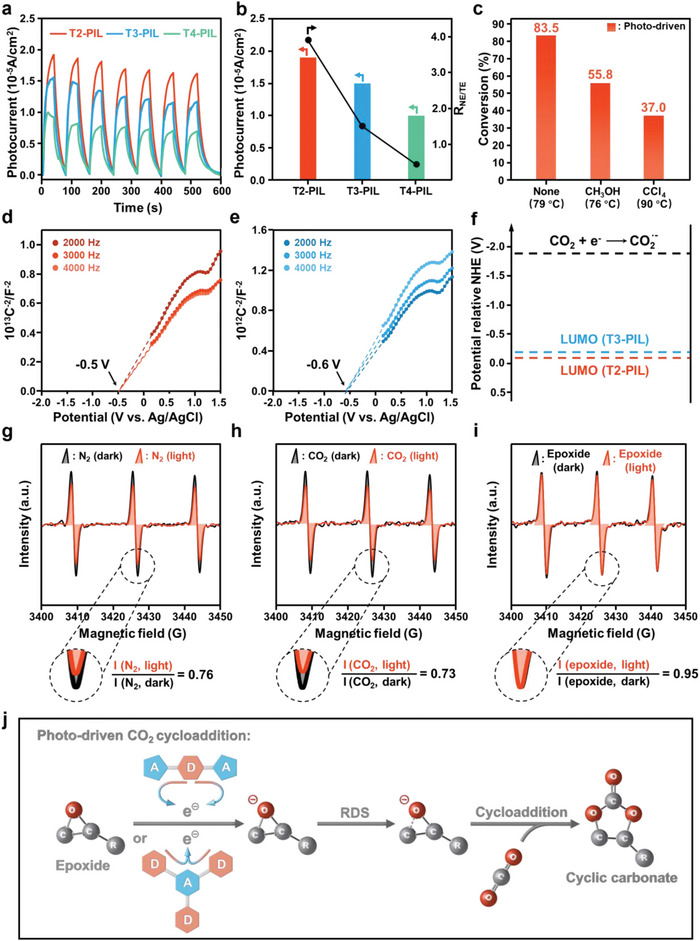
Mechanism study of the photo‐driven CO_2_ cycloaddition with D‐A type PILs. a) The photocurrent responses of T2‐PIL, T3‐PIL, and T4‐PIL. b) Photocurrents and the corresponding *R*_NE/TE_ of T2‐PIL, T3‐PIL, and T4‐PIL. c) Quenching experiments with different sacrificial agents using T2‐PIL as catalyst. Mott–Schottky curves of d) T2‐PIL and e) T3‐PIL. f) LUMO position of T2‐PIL and T3‐PIL and the redox potential of CO_2_/CO_2_^−^. EPR spectra of T2‐PIL under g) N_2_ atmosphere, h) CO_2_ atmosphere, and i) with glycidyl phenyl ether in N_2_ atmosphere. j) Proposed mechanism of photo‐driven CO_2_ cycloaddition reaction by D‐A PILs.

To study the photo‐induced charge separation and transfer behavior in the PILs with different D‐A segments, photocurrent tests and nanosecond transient absorption (TA) spectra are conducted. Among the PILs, T2‐PIL exhibits the highest photocurrent under irradiation, followed by T3‐PIL and then T4‐PIL, indicating the strongest charge separation efficiency in T2‐PIL (**Figure** **5**a). Moreover, the photocurrent intensity of the PILs totally follows the trend of the *R*_NE/TE_ in photo‐driven CO_2_ cycloaddition, suggesting that photo‐induced charges play vital roles in this reaction (Figure 5b). The lifetimes of the photogenerated carriers in PILs can be obtained from the TA spectra. Similarly, photogenerated carriers with the longest lifetime generate in T2‐PIL, followed by T3‐PIL and then T4‐PIL (Figure S15, Supporting Information).^[^ ^49^ ^]^ These results confirm that the photo‐induced charge separation in the PILs greatly improves their catalytic performance in photo‐driven CO_2_ cycloaddition. Quenching experiments are further conducted to further determine whether photogenerated electrons or holes play a role. First, methanol (CH_3_OH) is used as a hole sacrificial agent. When CH_3_OH is added to the photo‐driven catalytic system, the conversion rate of glycidyl phenyl ether reduces from 83.5% to 55.8% (Figure 5c). When carbon tetrachloride (CCl_4_) is added to the catalytic system as an electron sacrificial agent,^[^ ^50^ ^]^ the system temperature increases from 79 to 90 °C, but there is a more pronounced drop in conversion rate (from 83.5% to 37.0%). Thus the improved catalytic performance of T2‐PIL in the photo‐driven CO_2_ cycloaddition can be ascribed mainly to the photogenerated electrons.

The above results confirm that the charge separation and electron transfer occur in these D‐A‐based PILs. Another argument is whether the electrons transfer to CO_2_ or epoxides during the reaction. The positive slopes of the Mott–Schottky curves of the T2‐PIL and T3‐PIL indicate their n‐type semiconductor features, in which the majority carriers are electrons (Figure 5d,e).^[^ ^51^ ^]^ Their lowest unoccupied molecular orbital (LUMO) potentials are −0.09 and −0.19 V (vs Normal Hydrogen Electrode (NHE) at pH = 0) according to the Mott–Schottky plots, respectively. As the photoreduction process of CO_2_ to CO_2_^·−^ requires a very negative equilibrium potential of −1.9 V (vs NHE at pH = 0), neither T2‐PIL nor T3‐PIL have enough driving force for this process (Figure 5f).^[^ ^52^ ^]^ This suggests that the photogenerated electrons cannot transfer to CO_2_ during the reaction. To further explore the transfer path of the photogenerated electrons, the EPR spectra of T2‐PIL and T3‐PIL are tested at different conditions (Figure 5g–i and Figure S16, Supporting Information). 2,2,6,6‐Tetramethylpiperidine‐1‐oxyl (TEMPO) is used as the EPR spin label.^[^ ^53^ ^]^ A distinct triplet peak, which is the signal of unpaired electrons in TEMPO,^[^ ^54^ ^]^ can be seen at all tested conditions. In N_2_ atmosphere, the spectral intensities (*I*) significantly reduce after irradiation, and the calculated *I* (N_2_, light)/*I* (N_2_, dark) is 0.76 (Figure 5g). This decrease results from that electrons generate in T2‐PIL under illumination are captured by TEMPO, and partial TEMPO form the negative ion species TEMPO^−^. TEMPO^−^ has no unpaired electrons and is EPR silent. A similar intensity reduction is observed when the EPR test is performed in CO_2_ atmosphere, with an *I* (CO_2_, light)/*I* (CO_2_, dark) of 0.73 (Figure 5h). This confirms that the photogenerated electrons can't transfer to CO_2_, but are captured by TEMPO. In contrast, the intensity of the triplet peak remains unchanged before and after illumination when glycidyl phenyl ether is added (Figure 5i). This result indicates that the photogenerated electrons transfer to the epoxide and thus TEMPO can still maintain the content of unpaired electrons. The same trend can also be observed in the EPR spectra of T3‐PIL (Figure S16, Supporting Information).

Up to now, it can be concluded that during the photo‐driven CO_2_ cycloaddition, the photo‐induced charge separation in PILs is facilitated by the D‐A segments. The photogenerated electrons subsequently transfer to the epoxides, resulting in the polarization of the epoxides to accelerate the rate‐determining step. As a result, excellent catalytic performance is achieved in photo‐driven CO_2_ cycloaddition (Figure 5j).

## 3 Conclusions

We rationally design and synthesize a series of PILs with D‐A segments for photo‐driven CO_2_ cycloaddition in the absence of metal species, cocatalysts and solvents. The conversion of glycidyl phenyl ether in photo‐driven reaction can reach 4.9 times that of the thermal‐driven reaction at the same temperature, with the ratio of nonthermal effect to thermal effect as high as 3.9. A combination of DFT calculations and a series of mechanism experiments verify that the photogenerated electrons in PILs transfer to the epoxides and accelerate their ring‐opening, thus promoting the CO_2_ cycloaddition. This work proves that the photoelectric properties of PILs can be optimized by introduction of D‐A segments, which provides new insights into catalysts design for photo‐driven CO_2_ cycloaddition.

## 4 Experimental Section

### DFT Calculations

The photo‐excitation of different segments were evaluated by using a combination of DFT for the ground states and TD‐DFT for the excited state. The DFT calculations were carried out using the B3LYP hybrid Exchange–Correlation functional with the D3 version of Grimme's dispersion as implemented in Gaussian 16.^[^ ^55^, ^56^, ^57^, ^58^, ^59^ ^]^ The ground‐state geometry optimization calculation employed the 6‐31G(d, p) basis set. The TD‐DFT calculations were performed with CAM‐B3LYP method instead of B3LYP, as the CAM‐B3LYP functional is more reliable for the excitation energies calculation.^[^ ^28^, ^60^ ^]^

The hole and electron distribution are calculated with Equations (1)–(6) using the Multiwfn code

(1) $$\rho^{\mathrm{hole}}\left(r\right)=\rho_{\mathrm{loc}}^{\mathrm{hole}}\left(r\right)+\rho_{\mathrm{cross}}^{\mathrm{hole}}\left(r\right)$$

(2) $$\rho_{\mathrm{loc}}^{\mathrm{hole}}\left(r\right)=\sum_{i\to a}{\left(w_{i}^{a}\right)}^{2}\varphi_{i}\varphi_{i}-\sum_{i\leftarrow a}{\left(w_{i}^{\prime a}\right)}^{2}\varphi_{i}\varphi_{i}$$

(3) $$\rho_{\mathrm{cross}}^{\mathrm{hole}}\left(r\right)=\sum_{i\to a}\sum_{j\neq i\to a}w_{i}^{a}w_{j}^{a}\varphi_{i}\varphi_{j}-\sum_{i\leftarrow a}\sum_{j\neq i\leftarrow a}w_{i}^{\prime a}w_{j}^{\prime a}\varphi_{i}\varphi_{j}$$

(4) $$\rho^{\mathrm{ele}}\left(r\right)=\rho_{\mathrm{loc}}^{\mathrm{ele}}\left(r\right)+\rho_{\mathrm{cross}}^{\mathrm{ele}}\left(r\right)$$

(5) $$\rho_{\mathrm{loc}}^{\mathrm{hole}}\left(r\right)=\sum_{i\to a}{\left(w_{i}^{a}\right)}^{2}\varphi_{a}\varphi_{a}-\sum_{i\leftarrow a}{\left(w_{i}^{\prime a}\right)}^{2}\varphi_{a}\varphi_{a}$$

(6) $$\rho_{\mathrm{cross}}^{\mathrm{hole}}\left(r\right)=\sum_{i\to a}\sum_{i\to b\neq a}w_{i}^{a}w_{i}^{b}\varphi_{a}\varphi_{a}-\sum_{i\leftarrow a}\sum_{i\leftarrow b\neq a}w_{i}^{\prime a}w_{i}^{\prime b}\varphi_{a}\varphi_{a}$$

where *ρ* ^hole^(*r*) and *ρ* ^ele^(*r*) stand for the density distribution of hole and electron, respectively, *r* is the distance, *φ* is the orbital wave function, *φ_i_* or *φ_j_* is the occupied orbital, and *φ_a_* or *φ_b_* is the unoccupied orbital. Therefore, *i*→*a* represents excitation configuration, *i*←*a* represents de excitation configuration. Hole distribution and electron distribution are divided into local term and cross term. The local term is generally dominant, reflecting the contribution of the configuration function itself, and the cross term cannot be ignored, otherwise the quantification is inaccurate, which reflects the influence of the coupling between the configuration functions on the hole and electron distribution.

S and *E*_C_ indexes stand for the overlap integral of electron–hole distribution and the coulomb attraction energy between the hole and electron, respectively. The S and *E*_C_ are calculated with Equations (7) and (8) using Multiwfn code

(7) $$S=\int \min\left[\rho^{\mathrm{hole}}\left(r\right),\rho^{\mathrm{ele}}\left(r\right)\right]dr$$

(8) $$E_{C}=\;\int \int \;\frac{\rho^{\mathrm{hole}}\left(r_{1}\right)\rho^{\mathrm{ele}}\left(r_{2}\right)}{\left|r_{1}-r_{2}\right|}dr_{1}dr_{2}$$

where *ρ* ^hole^(*r*) and *ρ* ^ele^(*r*) stand for the density distribution of hole and electron, respectively. The detailed settings and process can be found in part 4.18 “Electron excitation analysis” in the manual of Multiwfn code.

### Synthesis of Electron Donor–Acceptor Segment 2,4,6‐Tris(4‐(bromomethyl)phenyl)‐1,3,5‐triazine

2,4,6‐Tris(4‐(bromomethyl)phenyl)‐1,3,5‐triazine (DA3) was synthesized by the method reported in the literature (Figure S17, Supporting Information).^[^ ^62^ ^]^ 4‐Cyanobenzyl bromide (2 g, 10.2 mmol) was added to the reaction tube, closed with a rubber stopper. Then, the reaction tube was evacuated and filled with nitrogen (N_2_). The reaction tube was cooled to 0 °C in a cryogenic reactor. Trifluoromethanesulfonic acid (2.7 mL, 33.8 mmol) was carefully injected into the reaction tube at 0 °C. Then the reaction tube was removed from the cryogenic reactor and stirred at room temperature for 13 h. After stirring, the orange‐yellow liquid in the reaction tube was introduced into ice water in a 250 mL beaker, and the appearance of white precipitate could be observed. Then 6 mL concentrated ammonia water was added to the beaker to neutralize the excess trifluoromethanesulfonic acid. The white solid was obtained by suction filtration and washed with acetone and water, and then placed in a vacuum drying oven at 60 °C overnight. The raw material and the obtained product were analyzed by IR spectroscopy tests. It can be seen from the results (Figure S17b,c, Supporting Information) that there is no absorption peak at 2226 cm^−1^ attributed to the —C≡N functional group in the IR spectrum of the product. At the same time, absorption peaks at 1518 and 1371 cm^−1^ attributed to the triazine group appeared, indicating the successful preparation of the product. ^1^H NMR (600 MHz, CDCl_3_, *δ*): 8.73 (d, *J* = 8.3 Hz, 6H; Ar H), 7.60 (d, *J* = 8.3 Hz, 6H; Ar H), 4.59 (s, 6H; CH_2_).

### Synthesis of Poly(ionic liquid) T2‐PIL

T2‐PIL was synthesized by Menshutkin reaction using two D‐A segments, 1‐(4‐imidazol‐1‐ylphenyl)imidazole and 2,4,6‐tris(4‐(bromomethyl)phenyl)‐1,3,5‐triazine. Considering the complexity of the expressions, they are abbreviated as DA1 and DA3, respectively (Table S1, Supporting Information). DA1 (0.2102 g, 1.0 mmol) and DA3 (0.3921 g, 0.67 mmol) were added to a reaction tube, followed by a magneton and N,N‐Dimethylformamide (DMF) (10 mL). The reaction tube was sealed with a rubber plug and vacuumed. Then nitrogen was injected in the system. After being heated in oil bath at 80 °C for 4 h and cooled to room temperature, the precipitate was filtered and the unreacted reagents were washed with DMF and methanol. The poly(ionic liquid) T2‐PIL was then obtained by vacuum drying it at 60 °C for 12 h.

### Synthesis of Poly(ionic liquid) T3‐PIL

T2‐PIL was synthesized by Menshutkin reaction using two D‐A segments, 1,4‐di(pyridine‐4‐yl)benzene and 2,4,6‐tris(4‐(bromomethyl)phenyl)‐1,3,5‐triazine. Considering the complexity of the expressions, they are abbreviated as DA2 and DA3, respectively (Table S1, Supporting Information). DA2 (0.2323 g, 1.0 mmol) and DA3 (0.3921 g, 0.67 mmol) were added to a reaction tube, followed by a magneton and DMF (10 mL). The reaction tube was sealed with a rubber plug and vacuumed. Then nitrogen was injected in the system. After being heated in an oil bath at 80 °C for 4 h and cooled to room temperature, the precipitate was filtered and the unreacted reagents were washed with DMF and methanol. The poly(ionic liquid) T3‐PIL was then obtained by vacuum drying it at 60 °C for 12 h. Other poly(ionic liquid)s used in this work were synthesized under the same reaction conditions using the corresponding building blocks (Table S1, Supporting Information).

### Synthesis of CTF‐2‐400

CTF‐2‐400 was synthesized by the ionothermal method as previously reported.^[^ ^62^ ^]^ Typically, 2,6‐pyridinedicarbonitrile and zinc chloride were evenly mixed in a glove box and the mixture is transferred into a Pyrex ampule. The ampoule tube was vacuumed with a vacuum pump and then it was sealed with an oxyhydrogen flame. The sealed ampoule tube was heated in a Muffle furnace at 400 °C for 20 h. The ampoule tube was cooled to room temperature and opened carefully. The resulting black solid was ground into black powder using a mortar. The black powder was then stirred in a solution of dilute hydrochloric acid for 12 h and in deionized water for 12 h to remove excess zinc chloride. The powder was filtered and vacuum dried at 120 °C for 12 h.

### In Situ DRIFTS

In situ DRIFTS measurement was carried out on a Bruker VERTEX 70v spectrophotometer. PILs were placed in the reaction chamber and sealed with a dome. First, the samples were pretreated with He stream (10 mL min^−1^) for 60 min at 80 °C. After cooling to room temperature, CO_2_ (10 mL min^−1^) was pumped into the chamber for 5 min. At last, CO_2_ was cut off and the samples stayed in static CO_2_ atmosphere. Spectra were collected at different time intervals in the above processes.

### Catalytic Tests

The photo‐driven catalytic reactions were carried out in a transparent glass flask (4 mL). Only catalysts and epoxides (14 mmol) were added to the flask, then vacuumized and connected with a CO_2_ balloon. For the heterogeneous catalysts, the amount was 50 mg. For the homogeneous catalysts, the amount of OmimBr‐IL and TBAB are 386 and 451 mg, respectively. A 300 W Xenon lamp (*λ* = 200–1000 nm) was used as the only energy source to drive the cycloaddition reaction for 12 h. Sufficient contact between catalyst and reactant was achieved by magnetic stirring. During the light irradiation, the temperature of the solution was recorded by an IR camera every 2 h and the average temperature was used as the reaction temperature in thermal‐driven catalytic reaction.

The thermal‐driven cycloaddition reaction took place in the same flask with photo‐driven reaction. Only catalysts and epoxides were added to the flask, which is then vacuumized and filled with a CO_2_ balloon. The flask was placed in an oil bath and heated to the set temperature with magnetic stirring. A covering was placed around the flask to avoid any light from the laboratory.

Ratios of the nonthermal effect to thermal effect (*R*_NE/TE_) were obtained by (photo‐driven conversion (%) − thermal‐driven conversion (%))/thermal‐driven conversion (%).^[^ ^43^ ^]^

### Quenching Experiments

The quenching experiments were carried out in a transparent glass flask. Besides the catalyst (T2‐PIL, 50 mg) and the epoxide (glycidyl phenyl ether, 14 mmol, 1.89 mL), an additional sacrificial agent (methanol (2.5 mmol, 0.1 mL) or carbon tetrachloride (2.5 mmol, 0.24 mL)) was added to the flask. The flask was then vacuumized and connected with a CO_2_ balloon. A 300 W Xenon lamp (*λ* = 200–1000 nm) was used to drive the cycloaddition reaction. The current of the lamp was set to 15 A. Sufficient contact between catalyst and reactant was achieved by magnetic stirring. During the light irradiation, the temperature of the solution was recorded by an IR camera every 2 h.

## Conflict of Interest

The authors declare no conflict of interest.

## Supporting information

Supporting Information

## Data Availability

The data that support the findings of this study are available from the corresponding author upon reasonable request.
