# Supplementary material for: Poly(ionic liquid)s for Photo‐Driven CO2 Cycloaddition: Electron Donor–Acceptor Segments Matter
Source: Adv Sci (Weinh). 2023 Jan 15;10(8):2206687. doi: 10.1002/advs.202206687 (PMC10015876; doi:10.1002/advs.202206687)
Supplement: Supplementary file 1 — Supporting Information [file ADVS-10-2206687-s001.pdf]

ADVANCED SCIENCE
Open Access

Supporting Information

for *Adv. Sci.*, DOI 10.1002/advs.202206687

Poly(ionic liquid)s for Photo-Driven CO_2 Cycloaddition: Electron Donor–Acceptor Segments Matter

Xu Fang, Li Yang, Zhangben Dai, Die Cong, Daoyuan Zheng, Tie Yu, Rui Tu, Shengliang Zhai, Junxia Yang, Fengling Song, Hao Wu, Wei-qiao Deng and Chengcheng Liu**

Supporting Information

Poly(ionic liquid)s for photo-driven CO_2 cycloaddition: Electron donor-acceptor segments matter

Xu Fang[†]*, Li Yang*[†]*, Zhangben Dai, Die Cong, Daoyuan Zheng, Tie Yu, Rui Tu, Shengliang Zhai, Junxia Yang, Fengling Song, Hao Wu, Wei-qiao Deng,* and Chengcheng Liu**

Materials and equipments: All the chemicals in this work were used directly as received. Epichlorohydrin (99.5%) was supplied by Shanghai Chemical Reagent Company of the Chinese Medicine Group. Glycidyl phenyl ether (99%) was supplied by Aladdin Biochemical Technology Co., Ltd. 1,4-di(pyridine-4-yl)benzene (97%), 4,4'-bipyridine (98%) and 4-cyanobenzyl bromide (98%) were supplied by Bidepharm Co., Ltd. 1-(4-imidazol-1-ylphenyl)imidazole (98%) was supplied by Yanshen Technology Co., Ltd. 1,3,5-tris(bromomethyl)benzene (98%), zinc chloride (99.9%), tetrabutylammonium bromide (TBAB, 99.0%), activated charcoal (99.7%) and epibromohydrin (98%) were supplied by Shanghai Macklin Biochemical Co., Ltd. Allyl glycidyl ether (99%) was supplied by Shanghai Acmec Biochemical Co., Ltd. Trifluoromethanesulfonic acid (99%+) was supplied by Adamas Reagent Co., Ltd. 2,6-pyridinedicarbonitrile (97%) was supplied by J&K Scientific Co. Ltd. 1-octyl-3-methylimidazolium bromide (OmimBr, 98%) was supplied by Qingdao Ionike Co. Ltd. N,N-dimethylformamide (DMF) and methanol were purchased from Shanghai Chemical Reagent Company of the Chinese Medicine Group. A 300W Xenon lamp (Lamp House R300-3J, Japan) was used to provide light radiation.

Materials characterization: TEM was performed on a FEI Technai G2 F20 field emission transmission electron microscope. Scanning electron microscopy (SEM) was performed on a FEI Nova Nanosem 450 field emission scanning electron microscope. Thermogravimetric analysis (TG) was performed on the samples under the nitrogen (N_2) atmosphere on a NETZSCH STA 449F5 analyzer with a heating rate of 10 °C/min. FT-IR were obtained on a Bruker VERTEX 70v FT-IR spectrophotometer using a KBr pellet. Solid-state ^{13}C NMR spectra were recorded on a Bruker AV400MHz NMR spectrometer. X-ray photoelectron spectroscopy (XPS) analysis is carried out on Thermo Scientific Xi+ multi-functional X-ray energy spectrometer. CO_2 temperature-programmed desorption (CO_2-TPD) measurements were performed on a Micromeritics AutoChem II chemisorption analyzer. Chromatography-mass spectrometry (GC-MS) analysis was performed on a Shimadzu GCMS-QP2010 SE instrument equipped with a SH-RXI-5SIL MS column. N_2-sorption and CO_2-sorption analysis were carried out on a Quantachrome autosorb IQ analyzer. UV-vis absorption spectra were acquired with a Shimadzu UV-2600i spectrophotometer, using $BaSO_4$ as a reference. Photocurrent measurements were carried out in a three-electrode cell on an electrochemical workstation (CHI 760E). The nanosecond transient absorption spectra were obtained at ambient conditions using an LFP 1000 spectrometer. Electron paramagnetic resonance (EPR) measurements were performed on a Bruker EMXnano electron paramagnetic resonance spectrometer. Infrared (IR) images were obtained by FLIR camera (FLIR E53). In-situ DRIFTS measurement were carried out on a Bruker VERTEX 70v spectrophotometer. Oxygen bomb-ion chromatography tests were performed on a HR-15 (Changsha Changxing Gaojiao Instrument and Equipment Co., Ltd) oxygen bomb calorimeter control box and a Thermo Scientific DIONEX ICS-1100 ion chromatograph.

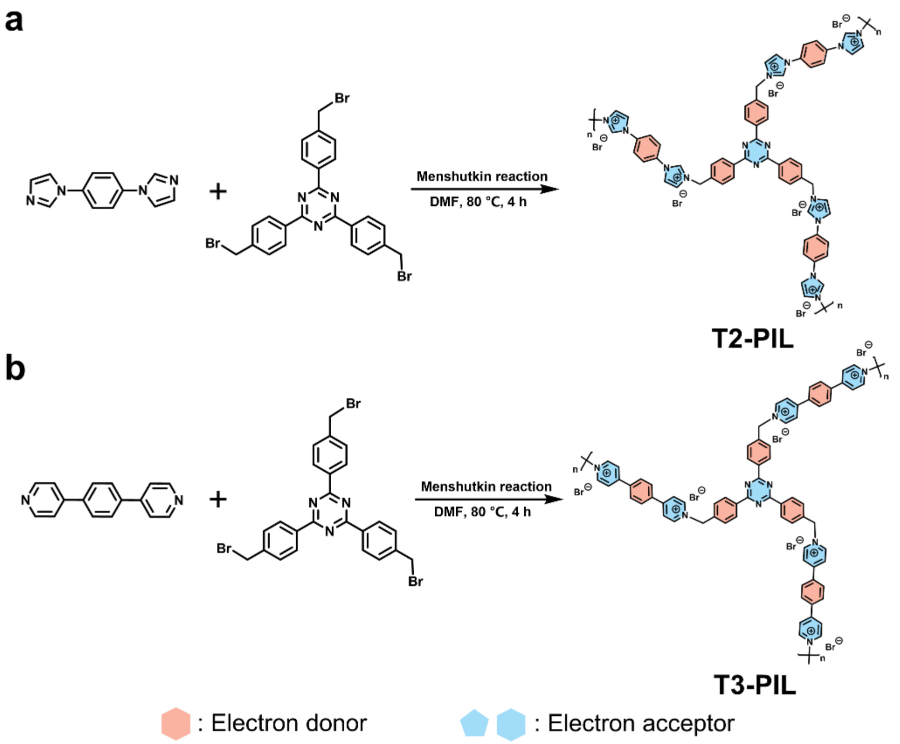

Figure S1. Synthetic schematics of (a) T2-PIL and (b) T3-PIL.

Table S1. Building blocks of different poly(ionic liquid)s

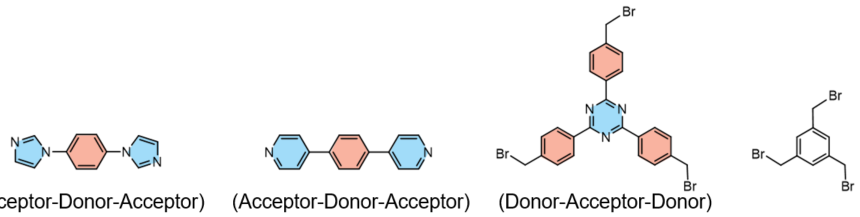

(Acceptor-Donor-Acceptor) DA1 | (Acceptor-Donor-Acceptor) DA2 | (Donor-Acceptor-Donor) DA3 | NDA1

Catalyst	Building blocks
T2-PIL	DA1 + DA3
T3-PIL	DA2 + DA3
NT2-PIL	DA1 + NDA1
NT3-PIL	DA2 + NDA1

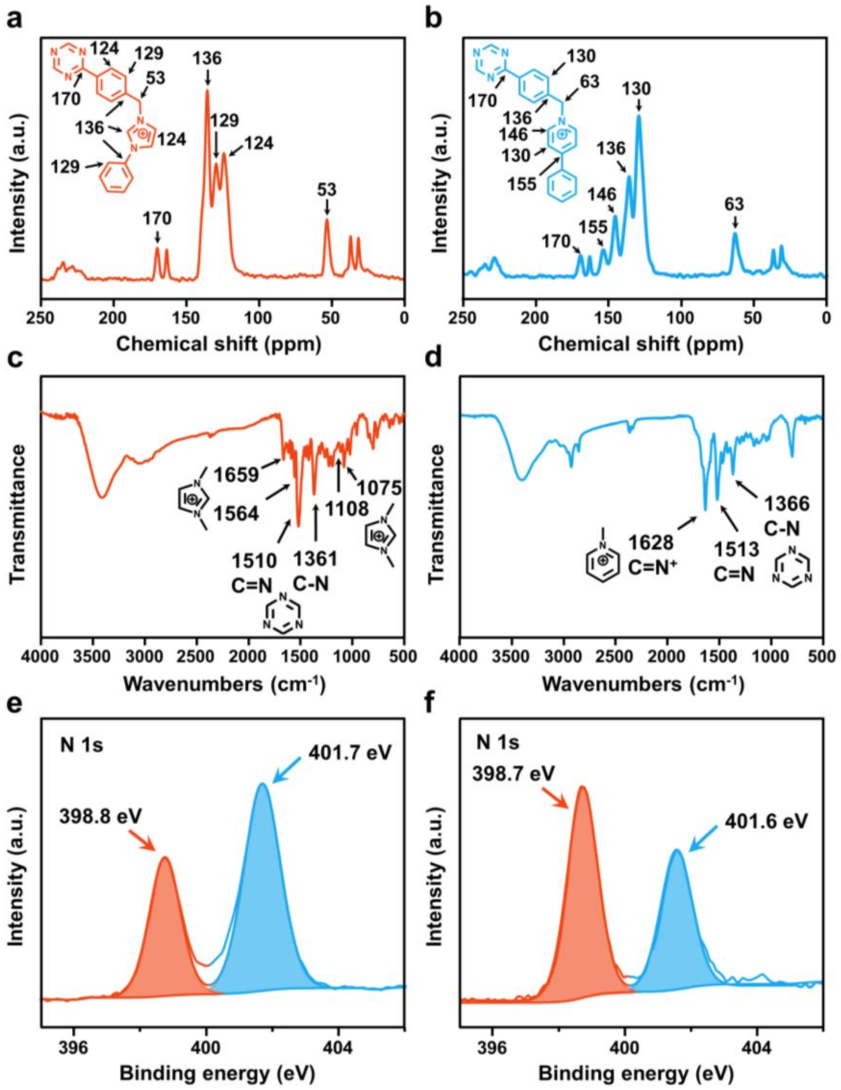

Figure S2. (a-b) Solid-state ^{13}C NMR of T2-PIL and T3-PIL. (c-d) FT-IR spectra of T2-PIL and T3-PIL. (e-f) XPS spectra of T2-PIL and T3-PIL.

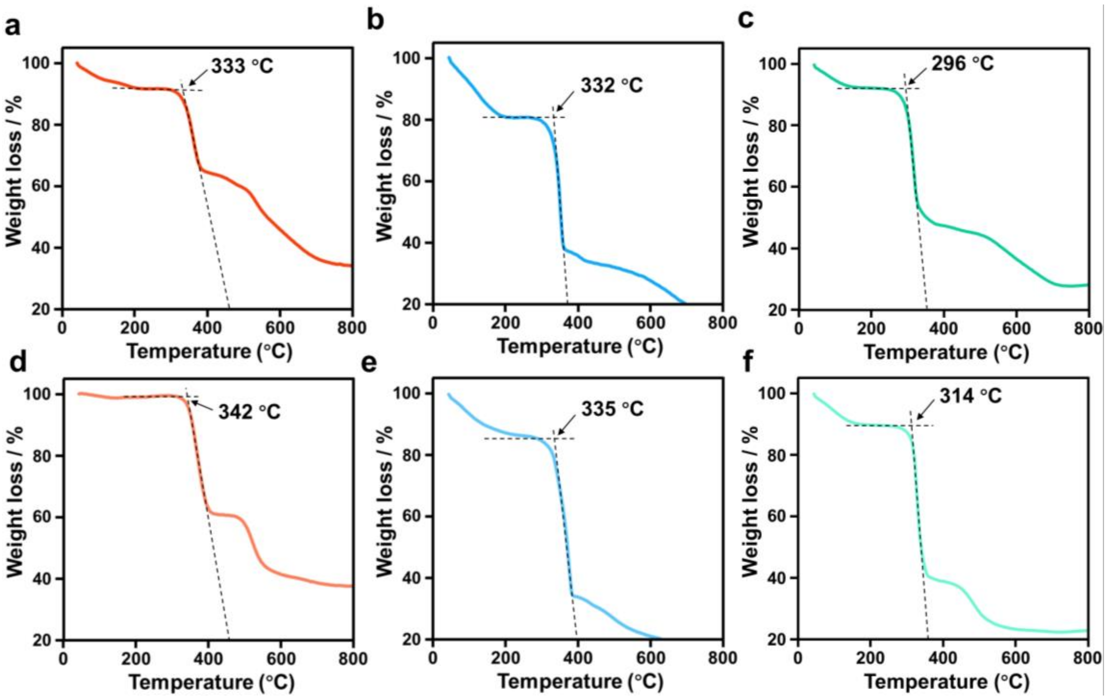

Figure S3. Thermogravimetric analysis (TGA) curves of (a) T2-PIL, (b) T3-PIL, (c) T4-PIL, (d) NT2-PIL, (e) NT3-PIL and (f) NT4-PIL.

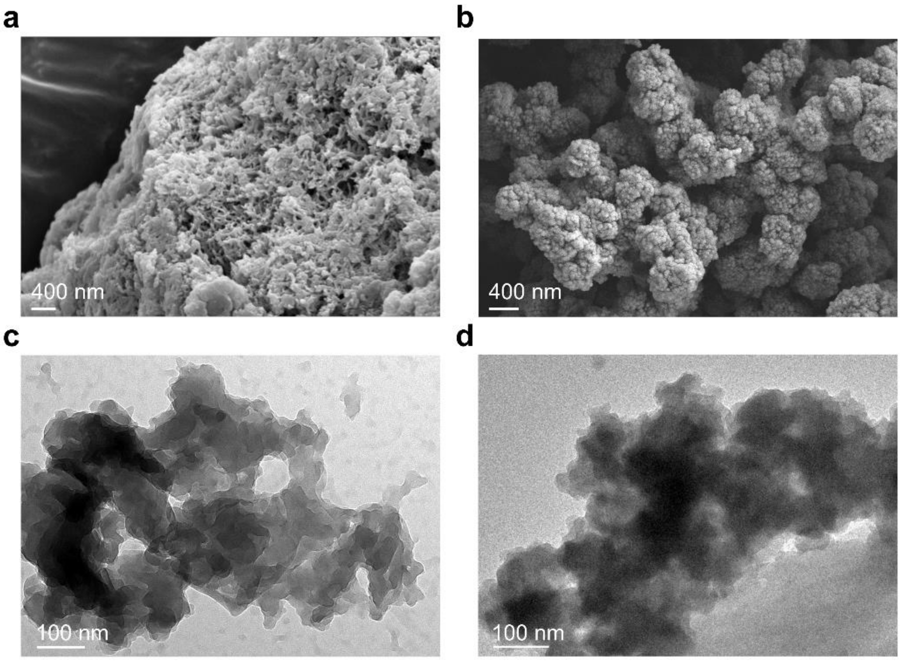

Figure S4. SEM images of (a) T2-PIL and (b) T3-PIL; TEM images of (c) T2-PIL and (d) T3-PIL.

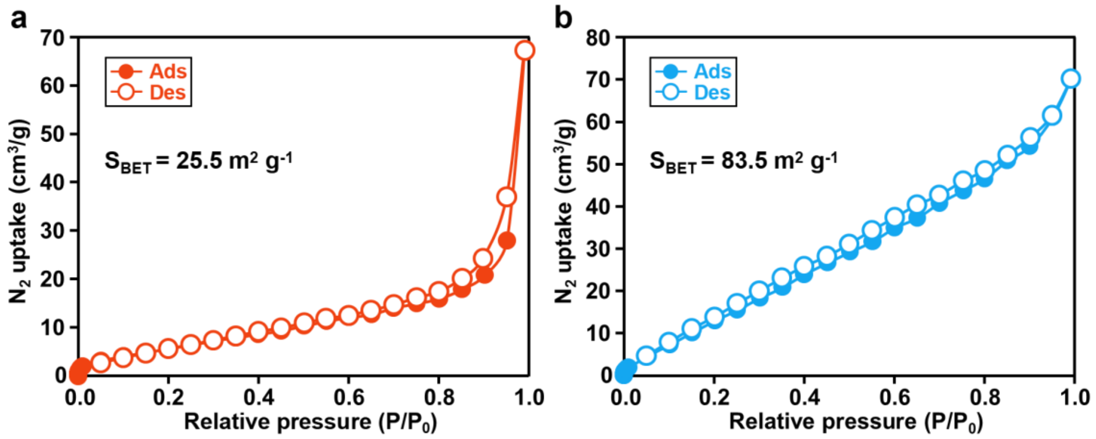

Figure S5. N_2 adsorption-desorption isotherms of (a) T2-PIL and (b) T3-PIL.

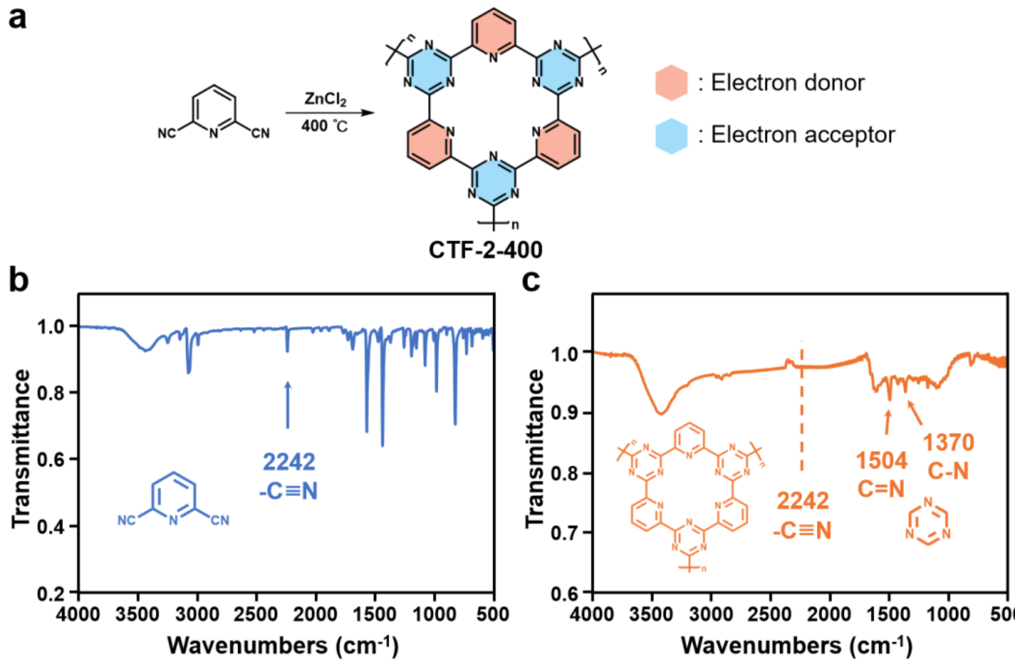

Figure S6. (a) Synthetic schematic of CTF-2-400. (b) IR spectrum of raw materials for synthesis of CTF-2-400. (c) IR spectra of CTF-2-400. In the FT-IR spectrum of the CTF-2-400, the disappearance of the absorption peak at 2242 cm^{-1} indicates that the reactants have been completely converted. The absorption peaks at 1504 cm^{-1} and 1370 cm^{-1} are attributed to the characteristic absorption peaks of triazine rings, which indicate that CTF-2-400 are successfully prepared by trimerization.

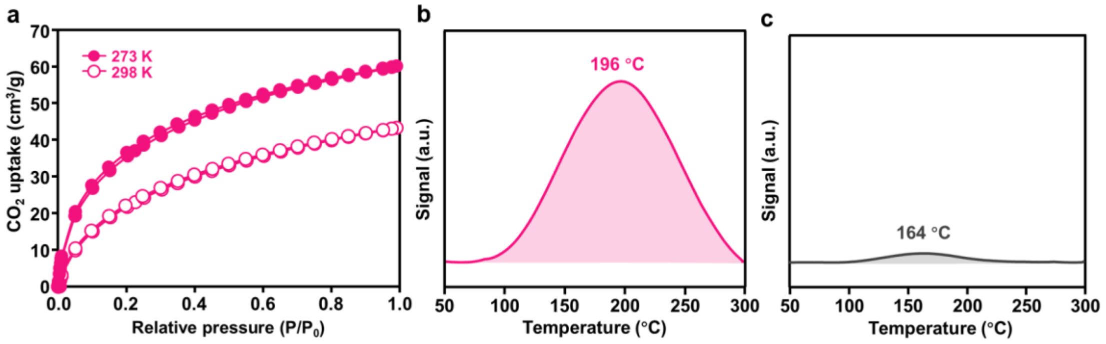

Figure S7. (a) CO_2 adsorption–desorption isotherms of CTF-2-400 at 273K and 298 K; Temperature-programmed CO_2 desorption measurement of (b) CTF-2-400 and (c) activated charcoal.

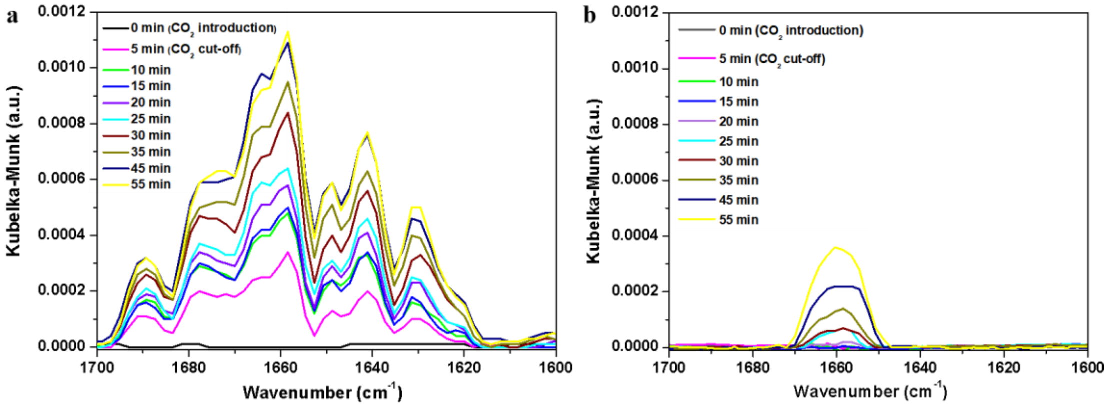

Figure S8. *In situ* DRIFTS spectra for CO_2 sorption on (a) T2-PIL and (b) NT2-PIL.

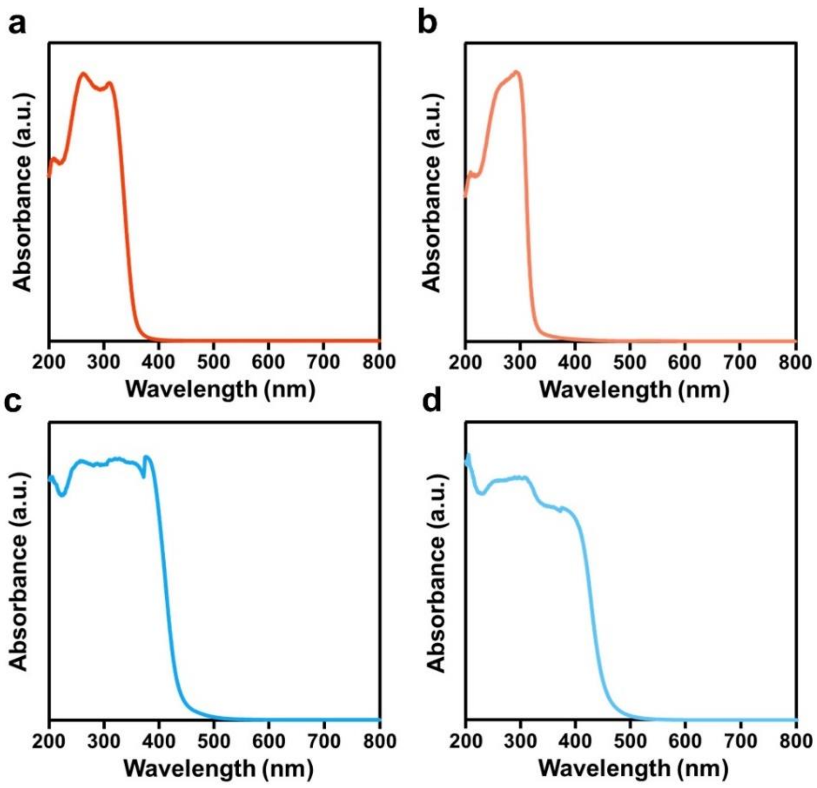

Figure S9. The UV-Vis spectra of (a) T2-PIL (b) NT2-PIL (c) T3-PIL and (d) NT3-PIL.

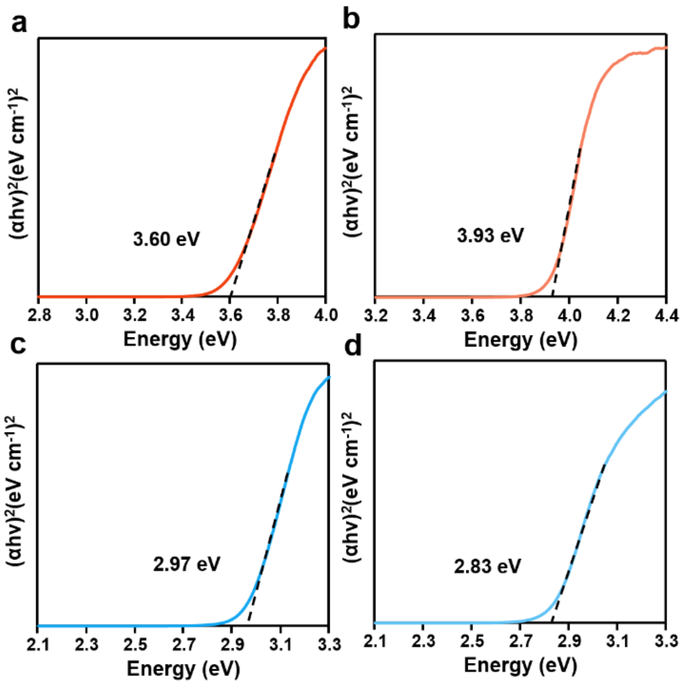

Figure S10. Band gap determination by Tauc plot for (a) T2-PIL, (b) NT2-PIL, (c) T3-PIL and (d) NT3-PIL.

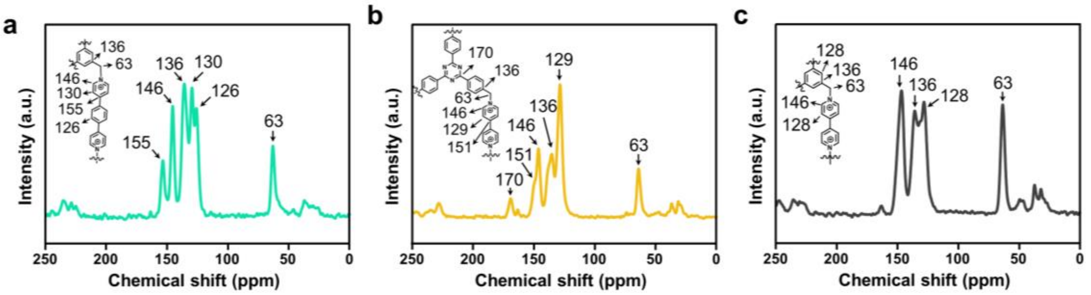

Figure S11. Solid-state ^{13}C NMR of (a) NT3-PIL, (c) T4-PIL and (d) NT4-PIL.

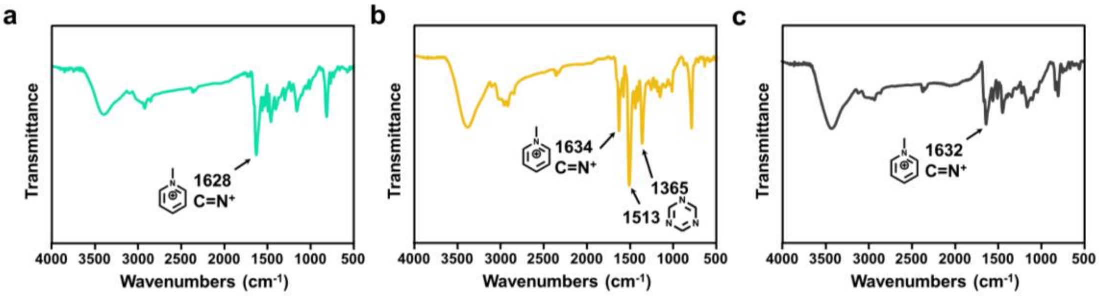

Figure S12. FT-IR spectra of (a) NT3-PIL, (b) T4-PIL and (c) NT4-PIL.

Table S2. Theoretical, XPS and oxygen bomb-ion chromatography (OB-IC) experimental element contents of PILs (H is excluded from the calculation).

Sample	Method	C (wt%)	N (wt%)	Br (wt%)
T2-PIL	Theoretical	58.0	14.5	27.5
	XPS	69.1	12.0	18.9
	OB-IC	/	/	20.9
T3-PIL	Theoretical	64.0	9.3	26.6
	XPS	64.9	9.0	26.1
	OB-IC	/	/	24.1
T4-PIL	Theoretical	59.1	10.6	30.3
	XPS	61.8	10.1	28.1
	OB-IC	/	/	24.7
NT3-PIL	Theoretical	58.4	6.2	35.4
	XPS	55.8	6.8	37.4
	OB-IC	/	/	33.8

Table S3. Comparison of catalytic performance of different metal-free heterogeneous catalysts for CO_2 cycloaddition at atmospheric pressure.

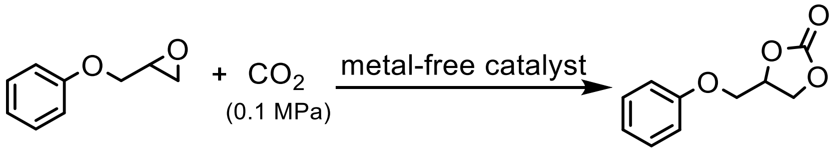

Catalyst	Temperature (℃)	Time (h)	Co-catalyst /solvent	Reaction rate (mmol g^{-1} h^{-1})	TOF (h^{-1})	Reference
**T2-PIL**	**79 (300 W 15 A)**	**12**	**None/None**	**19.5**	**5.90**	**This work**
PDMBr	120	12	None/None	8.03	3.09	S1
ImIP@TT-COF	120	24	None/None	12.3	Not provided	S2
PAD-3	110	8	None/None	5.94	2.58	S3
	90	24	None/None	1.88	0.82	
PIP-Bn-Cl	100	72	None/None	10.8	5.3	S4
COP-222	100	36	None/None	4.21	Not provided	S5
BGCNx	100	60	0.6 mol% KI/ None	3.30	Not provided	S6
Py-iPOP-1	100	72	None/None	0.49	Not provided	S7
IP 3	100	48	None/None	Not provided	0.36	S8
PP-Br	90	70	None/10 mL DMF	2.73	2.13	S9
POP-PA-NH_2	90	48	None/None	1.78	1.71	S10
PVIm6-SCD	80	24	None/None	6.94	0.81	S11
1D-UCP	80	48	1.0 mol% KI / None	3.07	Not provided	S12
VIPA-Br	80	48	None/None	1.03	Not provided	S13
COF-IL	80	48	None/None	0.81	0.49	S14
IMIN-Br-OH	80	72	None/None	0.66	0.16	S15
iPHCP-12	80	60	None/None	0.61	Not provided	S16

Table S4. Comparison of catalytic performance of different metal-based heterogeneous catalysts for photo-driven or thermal-driven CO_2 cycloaddition with glycidyl phenyl ether.

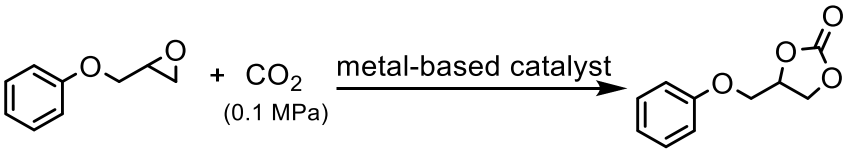

Catalyst	Temperature (℃)	Time (h)	Co-catalyst	Solvent	Reaction rate (mmol g^{-1} h^{-1})	Reference
**T2-PIL**	**79 (300 W Xe lamp)**	**12**	**None**	**None**	**19.5**	**This work**
SiWCo@GO-PEI	75 (808 nm laser)	4	3.9 mol% TBAB	None	10.3	S17
Ni-BNCNTs@ HMPs-NH_2	74 (300 W Xe lamp)	24	None	None	20.0	S18
ZnO/NCO-L	70 (300 W Xe lamp)	12	20 mol% TBAB	2 mL CH_3CN	0.44	S19
ZnS/NPC–2	65 (300 Xe lamp)	12	30 mol% TBAB	1 mL DMF	0.37	S20
Al–N–C	62 (400 W Xe lamp)	28	9.7 mol% TBAB	2 mL DMF	0.54	S21
FeTPyP	62 (300 W Xe lamp)	5	100 mol% TBAB	2 mL DMF	0.32	S22
Salen-Co(23 %)∈(Br^-) Etim-UiO-66	120	12	None	None	11.9	S23
(I-)Meim-UiO-66	120	24	None	None	6.3	S24
IL@ZIF-8(Zn/ Co)	100	24	None	None	4.5	S25
Rh-PMOF-1	100	24	0.2 mol% TBAB	None	4.1	S26
CN@MIL(400 ,30)	90	6	None	3 mL DMF	1.0	S27
FJI-C10	80	48	None	None	2.0	S28
Catalyst 1	80	12	10 mol% TBAB	None	2.5	S29
POM@ImTD-COF	80	24	1.6 mol% TBAB	1 mL DMF	2.8	S30
1·Mn	80	4	10 mol%	None	2.3	S31

			TBAB			
NPC-900	70	24	17 mol% TBAB	2 mL CH3CN	0.37	S32
polyILs@MIL-101	70	24	None	2 mL CH3CN	0.40	S33

Table S5. Comparison of catalytic systems for photo-driven CO_2 cycloaddition with different epoxides.

Catalyst	Temperature (℃)	Co-catalyst	Solvent	$R_{NE/TE}$	Reference
**T2-PIL**	**79**	**None**	**None**	**3.9**	**This work**
BiNbO4/5% r-GO	80	2 mol% TBAB	12 mL CH_3CN+MeOH	3.6	S34
Bi-PCN-224	50	11 mol% TBAB	None	1.5	S35
Al–N–C	62	9.7 mol% TBAB	2 mL DMF	0.67	S21
SiWCo@GO-PEI	75	3.9 mol% TBAB	None	0.40	S17
Zn SA-NC	65	66.7 mol% TBAB	None	0.17	S36
ZNC-800	82	0.5 mol% TBAB	None	0.13	S37
ZnS/NPC–2	65	30 mol% TBAB	1 mL DMF	0.032	S20
10.26 wt% PMo12@Zr-Fc	80	2 mol% TBAB	None	0.014	S38
MOF-1	57	2.5 mol% TBAB	None	0.0	S39
COF-PI-2	51	None	None	0.0	S40
Flower-like Co_2C	60	167 mol% TBAB	3 mL DMF	-0.014	S41
ZnO/NCO-L	70	20 mol% TBAB	2 mL CH_3CN	-0.084	S19

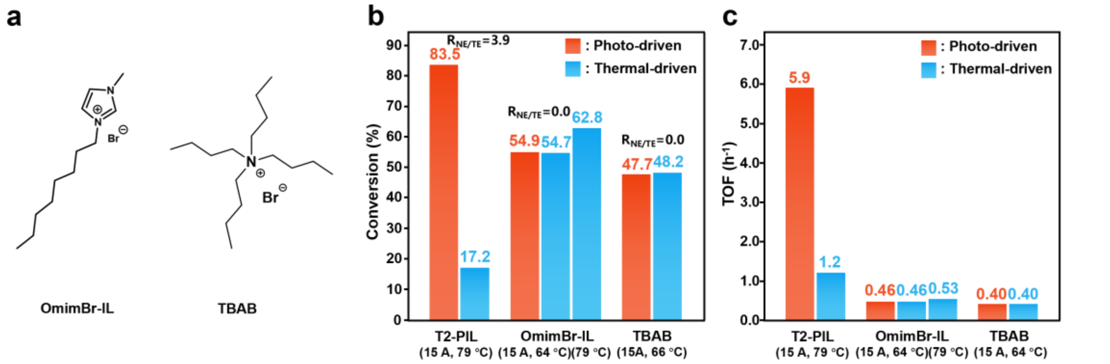

Figure S13. a) Structural formulas of homogeneous catalysts OmimBr-IL and TBAB. b) Comparison of the conversion rate of glycidyl phenyl ether. c) Comparison of TOF of glycidyl phenyl ether.

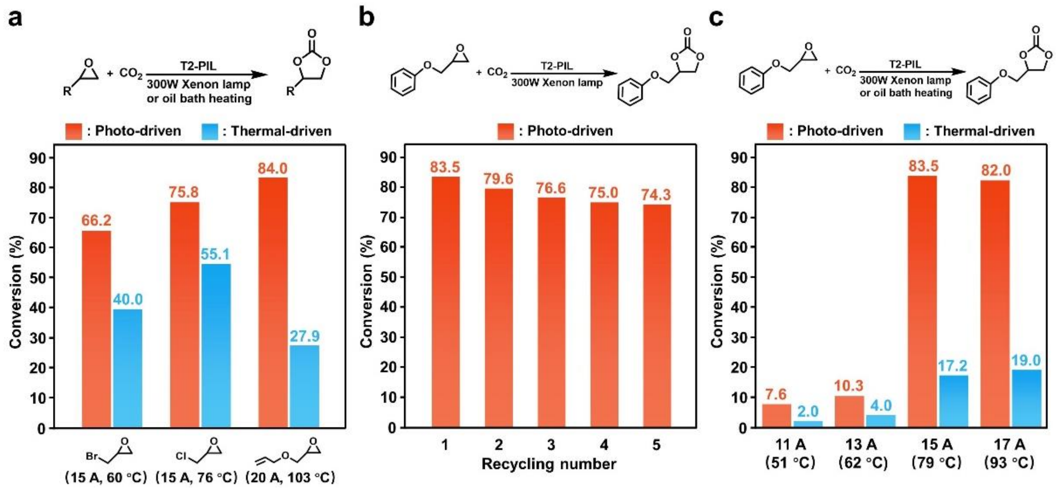

Figure S14. a) Catalytic compatibility tests of T2-PIL. b) Catalytic recyclability tests of T2-PIL. c) Comparison of conversion rates of CO_2 cycloaddition reaction at different light intensities and corresponding temperatures.

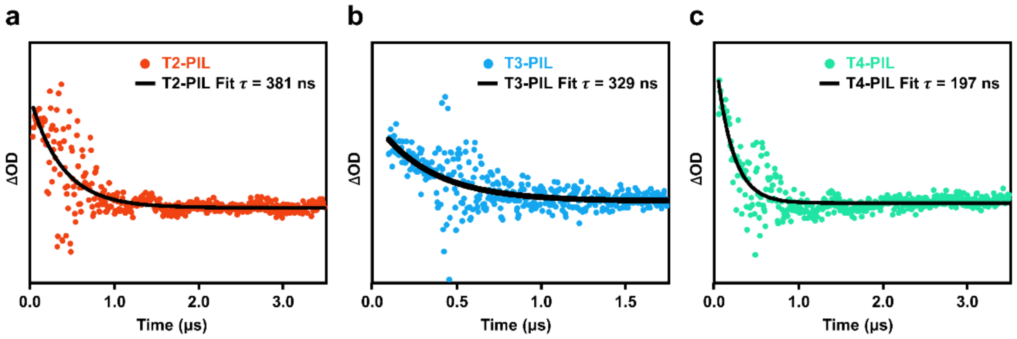

Figure S15. Nanosecond transient absorption (TA) spectra of the a) T2-PIL, b)

T3-PIL and c) T4-PIL.

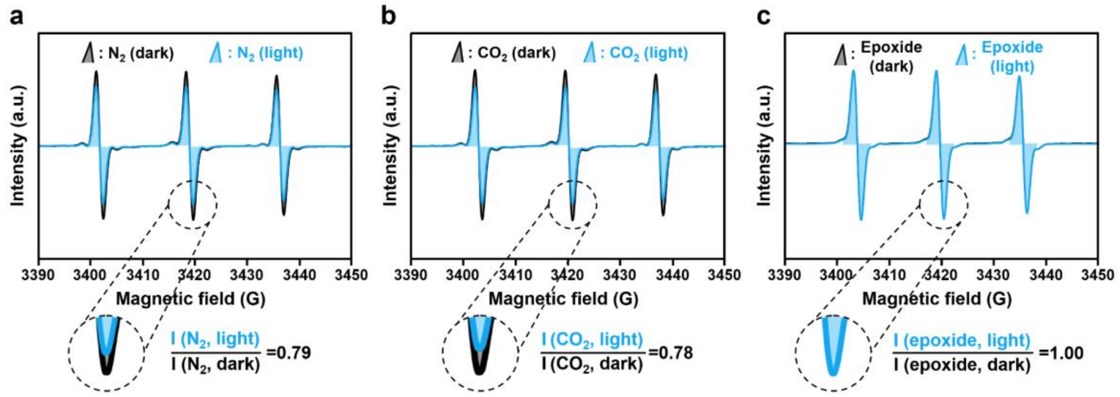

Figure S16. EPR spectra of T3-PIL under different conditions: a) N_2 atmosphere; b) CO_2 atmosphere, and c) added an epoxide (glycidyl phenyl ether) in N_2 atmosphere.

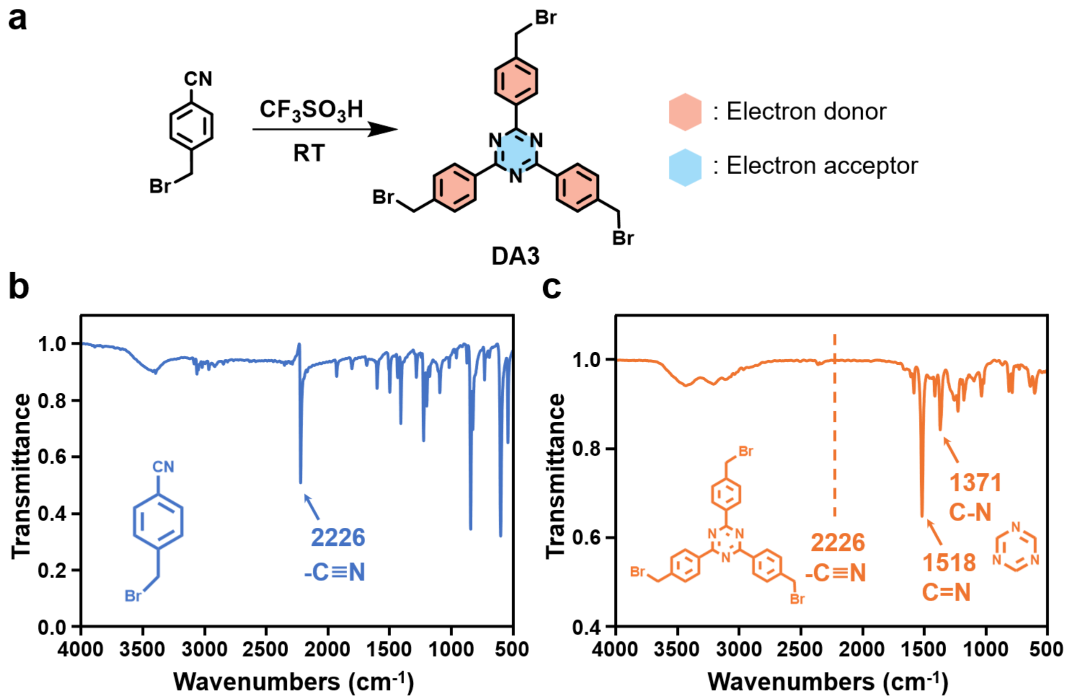

Figure S17. (a) Synthetic schematic of building block DA3. (b) IR spectrum of raw materials for synthesis of building block DA3. (c) IR spectrum of building block DA3.

Reference

[S1] X. Wang, Y. Zhou, Z. Guo, G. Chen, J. Li, Y. Shi, Y. Liu, J. Wang, *Chem. Sci.* **2015**, 6, 6916.

[S2] H. Zhong, J. Gao, R. Sa, S. Yang, Z. Wu, R. Wang, *ChemSusChem* **2020**, 13, 6323.

[S3] Z. Guo, X. Cai, J. Xie, X. Wang, Y. Zhou, J. Wang, *ACS Appl. Mater. Inter.* **2016**, 8, 12812.

[S4] Q. Sun, Y. Jin, B. Aguila, X. Meng, S. Ma, F. S. Xiao, *ChemSusChem* **2017**, 10, 1160.

[S5] S. Subramanian, J. Oppenheim, D. Kim, T. S. Nguyen, W. M. H. Silo, B. Kim, W. A. Goddard, C. T. Yavuz, *Chem* **2019**, 5, 3232.

[S6] H. Chand, P. Choudhary, A. Kumar, A. Kumar, V. Krishnan, *J. CO_2 Util.* **2021**, 51, 101646.

[S7] K. Liu, Z. Xu, H. Huang, Y. Zhang, Y. Liu, Z. Qiu, M. Tong, Z. Long, G. Chen, *Green Chem.* **2022**, 24, 136.

[S8]W. Zhong, F. D. Bobbink, Z. Fei, P. J. Dyson, *ChemSusChem* **2017**, 10, 2728.

[S9]Q. Zhang, S. Zhang, S. Li, *Macromolecules* **2012**, 45, 2981.

[S10] Z. Dai, Y. Bao, J. Yuan, J. Yao, Y. Xiong, *Chem. Commun.* **2021**, 57, 9732.

[S11] Y. Xie, J. Liang, Y. Fu, M. Huang, X. Xu, H. Wang, S. Tu, J. Li, *J. Mater. Chem. A* **2018**, 6, 6660.

[S12] Y. Hao, X. Yan, X. Liu, S. Qin, Z. Zhu, B. Panchal, T. Chang, *J. CO_2 Util.* **2022**, 56, 101867.

[S13] Z. Xu, K. Liu, H. Huang, Y. Zhang, Z. Long, M. Tong, G. Chen, *J. Mater. Chem. A* **2022**, 10, 5540.

[S14] L. Ding, B. Yao, F. Li, S. Shi, N. Huang, H. Yin, Q. Guan, Y. Dong, *J. Mater. Chem. A* **2019**, 7, 4689.

[S15] Y. Zhang, G. Chen, L. Wu, K. Liu, H. Zhong, Z. Long, M. Tong, Z. Yang, S. Dai, *Chem. Commun.* **2020**, 56, 3309.

[S16] Y. Zhang, K. Liu, L. Wu, H. Huang, Z. Xu, Z. Long, M. Tong, Y. Gu, Z. Qin, G.

Chen, *Dalton Trans.* **2021**, 50, 11878.

[S17] X. Chen, M. Wei, A. Yang, F. Jiang, B. Li, O. A. Kholdeeva, L. Wu, *ACS Appl. Mater. Inter.* **2022**, 14, 5194.

[S18] Y. Guo, W. Chen, L. Feng, Y. Fan, J. Liang, X. Wang, X. Zhang, J. *Mater. Chem. A* **2022**, 10, 12418.

[S19] C. Duan, M. Ding, Y. Feng, M. Cao, J. Yao, *Sep. Purif. Technol.* **2022**, 285, 120359.

[S20] F. Tang, L. Wang, L. Ma, Y. Fang, J. Huang, Y. Liu, *J. CO_2 Util.* **2021**, 45, 101431.

[S21] Q. Yang, H. Peng, Q. Zhang, X. Qian, X. Chen, X. Tang, S. Dai, J. Zhao, K. Jiang, Q. Yang, J. Sun, L. Zhang, N. Zhang, H. Gao, Z. Lu, L. Chen, *Adv. Mater.* **2021**, 33, e2103186.

[S22] H. Zhang, G. Zhai, L. Lei, C. Zhang, Y. Liu, Z. Wang, H. Cheng, Z. Zheng, P. Wang, Y. Dai, B. Huang, *J. Colloid Interface Sci.* **2022**, 625, 33.

[S23] T. T. Liu, J. Liang, R. Xu, Y. B. Huang, R. Cao, *Chem. Commun.* **2019**, 55, 4063.

[S24] J. Liang, R. P. Chen, X. Y. Wang, T. T. Liu, X. S. Wang, Y. B. Huang, R. Cao, *Chem. Sci.* **2017**, 8, 1570.

[S25] Y. Sun, X. Jia, H. Huang, X. Guo, Z. Qiao, C. Zhong, *J. Mater. Chem. A* **2020**, 8, 3180.

[S26] J. Liu, Y. Z. Fan, X. Li, Y. W. Xu, L. Zhang, C. Y. Su, *ChemSusChem* **2018**, 11, 2340.

[S27] F. Chen, K. Shen, L. Chen, Y. Li, *Sci. China Chem.* **2022**, 65, 1411.

[S28] J. Liang, Y. Q. Xie, X. S. Wang, Q. Wang, T. T. Liu, Y. B. Huang, R. Cao, *Chem Commun.* **2018**, 54, 342.

[S29] B. B. Lu, W. Jiang, J. Yang, Y. Y. Liu, J. F. Ma, *ACS Appl. Mater. Interfaces* **2017**, 9, 39441.

[S30] Y. Zhang, D. H. Yang, S. Qiao, B. H. Han, *Langmuir* **2021**, 37, 10330.

[S31] W. Jiang, J. Yang, Y. Y. Liu, S. Y. Song, J. F. Ma, *Chem. Eur. J.* **2016**, 22,

16991.

[S32] P. Ma, M. Ding, X. Liu, W. Rong, J. Yao, *Chem. Eng. Sci.* **2022**, 252, 117530.

[S33] M. Ding, H.-L. Jiang, *ACS Cata.* **2018**, 8, 3194.

[S34] M. Bakiro, S. Hussein Ahmed, A. Alzamly, *ACS Sustain. Chem. Eng.* **2020**, 8, 12072.

[S35] G. Zhai, Y. Liu, L. Lei, J. Wang, Z. Wang, Z. Zheng, P. Wang, H. Cheng, Y. Dai, B. Huang, *ACS Catal.* **2021**, 11, 1988.

[S36] L. Gong, J. Sun, Y. Liu, G. Yang, *J. Mater. Chem. A* **2021**, 9, 21689.

[S37] Y. Liu, Y. Chen, Y. Liu, Z. Chen, H. Yang, Z. Yue, Q. Fang, Y. Zhi, S. Shan, *J. Catal.* **2022**, 407, 65.

[S38] Z. Fang, Z. Deng, X. Wan, Z. Li, X. Ma, S. Hussain, Z. Ye, X. Peng, *Appl. Catal. B* **2021**, 296, 120329.

[S39] N. Sharma, S. S. Dhankhar, C. M. Nagaraja, *Microporous Mesoporous Mater.* **2019**, 280, 372.

[S40] L. Ding, B. Yao, W. Wu, Z. Yu, X. Wang, J. Kan, Y. Dong, *Inorg. Chem.* **2021**, 60, 12591.

[S41] Q. Guo, S. G. Xia, X. B. Li, Y. Wang, F. Liang, Z. S. Lin, C. H. Tung, L. Z. Wu, *Chem. Commun.* **2020**, 56, 7849.
